# Reccurent thrombus in the gigantic left atrium during effective anticoagulant therapy: case report

**DOI:** 10.1186/s12872-019-01279-1

**Published:** 2020-02-21

**Authors:** Lucia Masarova, Jan Novak, Martin Pesl, Jiri Ondrasek, Jiri Semenka, Eva Simarova, Roman Panovsky

**Affiliations:** 1 First Department of Cardioangiology, St. Anne’s University Hospital and Faculty of Medicine, Masaryk University, Brno, Czech Republic; 2 International Clinical Research Center (ICRC), St. Anne’s University Hospital, Brno, Czech Republic; 3 Second Department of Internal Medicine, St. Anne’s University Hospital and Faculty of Medicine, Masaryk University, Brno, Czech Republic; 4 Department of Physiology, Faculty of Medicine, Masaryk University, Brno, Czech Republic; 5 Department of Biology, Faculty of Medicine, Masaryk University, Brno, Czech Republic; 6 Center for Cardiovascular Surgery and Organ Transplantation, Brno, Czech Republic; 7 Department of Radiology, St. Anne’s University Hospital and Masaryk University, Brno, Czech Republic

**Keywords:** Cardiac magnetic resonance, Echocardiography, Atrial fibrillation, Recurrent thrombus, Gigantic left atrium, Anticoagulant therapy

## Abstract

**Background:**

Gigantic left atrium is defined in the current literature as an excessive dilatation of the left atrium above 65mm. Chronic mitral valve disease is associated with the development of thrombus in the left atrium in up to 19% of all cases of mitral insufficiency and appropriate treatment must be initiated to prevent thromboembolic events. In order to diagnose thrombi in the left atrium or left atrial appendage, various imaging methods may be used, including cardiac magnetic resonance.

**Case presentation:**

The case report describes a 73-year-old male who developed recurrent sessile thrombus on the posterior wall of the gigantic left atrium. A large thrombus was first detected following mitral valve surgery despite effective vitamin K antagonist anticoagulation therapy. Echocardiography and cardiac magnetic resonance were used within the diagnostic procedure and to monitor the treatment outcomes. Cardiac magnetic resonance was shown to be beneficial as it provided a more precise description of the intra-atrial masses located on the posterior left atrial wall, and in such situations, is of greater benefit than standard echocardiography. This led to the surgical removal of the intra-atrial mass; nevertheless, it was quickly followed by the recurrence of the thrombus. The anticoagulant therapy was adjusted and fortified by the introduction of acetylsalicylic acid and sequentially clopidogrel, but this also did not resolve the thrombus formation. Finally, employing a combination of rivaroxaban and clopidogrel resulted in partial thrombus regression. Therefore, various pathophysiological aspects of thrombus formation and used anticoagulation strategies are discussed.

**Conclusions:**

We describe a unique case of a recurrent thrombus located on the posterior wall of the gigantic left atrium. Cardiac magnetic resonance was shown to be beneficial in providing a more precise description of the intra-atrial masses located on the posterior left atrial wall as compared to standard echocardiographic examination. Development of a thrombus after mitral valve surgery despite effective anticoagulant therapy and its final resolution by introducing a combination of rivaroxaban and clopidogrel highlights the complex etiopathogenesis of thrombus formation. This supports the potential use of this combination in tailoring an individual personalized therapy for patients with recurrent atrial thrombi.

## Background

Gigantic left atrium (LA) is defined in the current literature as an excessive dilatation of the LA above 65mm as measured by echocardiography or cardiac magnetic resonance imaging (CMRI) and is most frequently caused by mitral insufficiency [1]. Chronic mitral valve disease is associated with the development of thrombus in the LA in up to 19% of all cases of mitral insufficiency [2, 3]. Gigantic LA is linked to the occurrence and persistence of atrial fibrillation (AF), but also to the development of a thrombus in the LA. Taken together, this significantly increases the risk of thrombotic events – such as stroke, acute limb ischemia or rarely even kidney infarctions [4]. In the case of stroke, AF is known to cause up to 20% of all ischemic strokes and thus proper rate control and especially anticoagulation therapy are necessary to help prevent thrombotic events [5]. In order to diagnose thrombi in the LA or left atrial appendage (LAA), various imaging methods may be used, including echocardiography [6], three-dimensional (3D) echocardiography or CMRI [7, 8]. A combination of the aforementioned methods may be needed for the final diagnosis and it also plays an important role in follow-up care and thrombus size description.

## Case presentation

The presented case report describes a 73-year-old male (born in 1944). There was no case-relevant information in his family history – his father died at a high age of 83 and his mother at 78 after a stroke. The patient experienced his first paroxysm of AF in 2001 (57 years old) and this was related to decompensated thyrotoxicosis (thyroid stimulating hormone <0.01 mU/l, free T4 32.40 pmol/l). He was also followed-up for hypertension and chronic obstructive pulmonary disease and was administered an appropriate therapy – theophylline, perindopril, betaxolol, furosemide, and due to the decompensated thyrotoxicosis, thyrosol. After the initial diagnosis, he underwent several successful electrical and pharmacological cardioversions that were sufficient until 2009 (65 years old), where no available means of cardioversion were able to end the arrhythmia. Thus, since 2009, his AF has been categorized as permanent and was indicated for full anticoagulation therapy using vitamin K antagonists (VKA, i.e. warfarin).

**Fig. 1:**
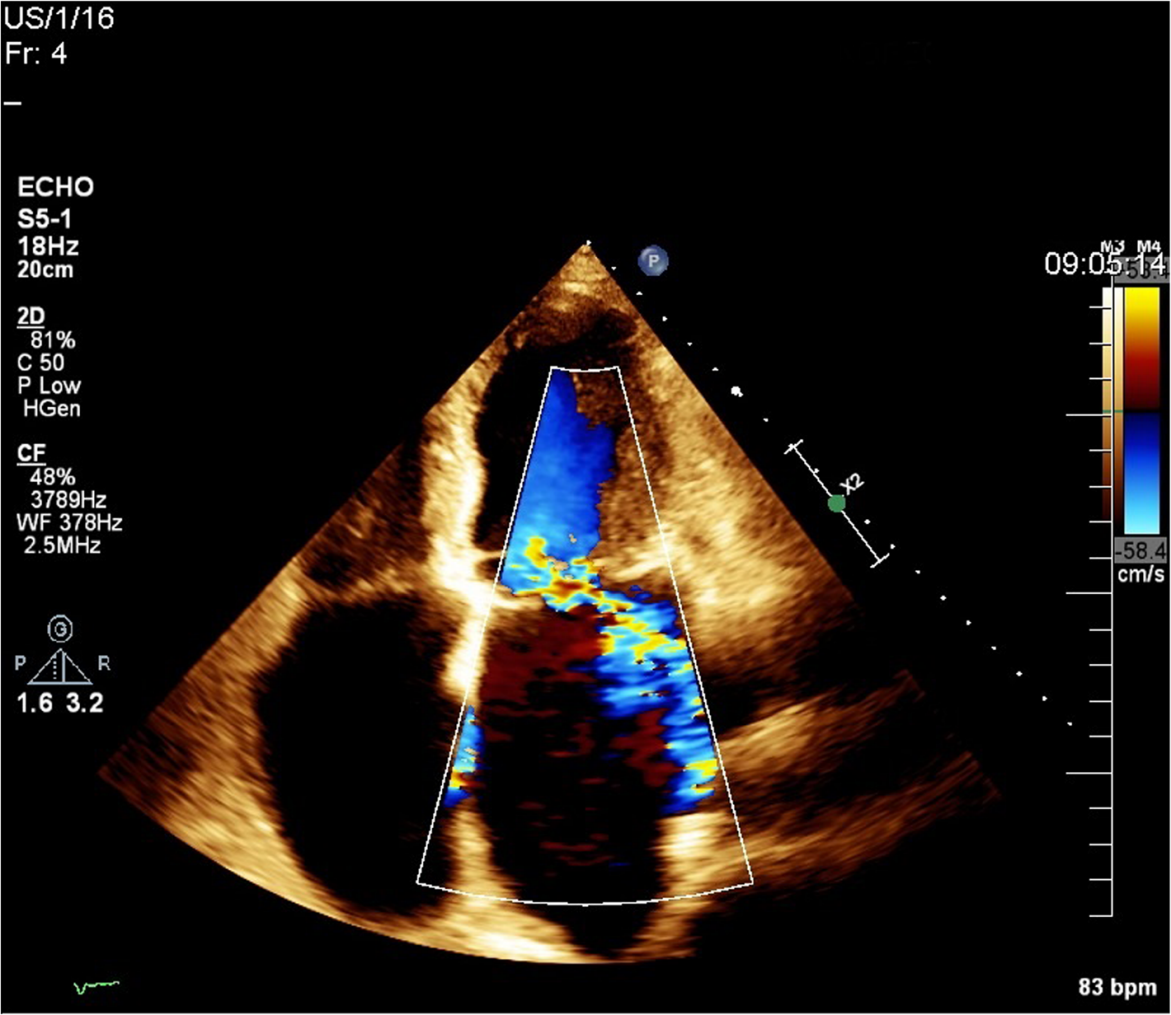
Echocardiogram on admission A four-chamber view showing giant LA, mitral annular dilatation with ruptured and flailed chordae tendinea of the anterior leaflet and significant mitral regurgitation

In 2017, after 8 years, he was admitted to the Cardiology Clinic. The precipitating reason for his admittance was progressive and exertional dyspnoea – New York Heart Association (NYHA) grade II-III. A transthoracic (TTE) and transoesophageal echocardiogram (TEE) revealed a giant LA sized 75x105mm, his left ventricular (LV) diastolic diameter was 60mm and the ejection fraction (EF) was 65%. Echocardiographists further described the presence of mitral annular dilatation with ruptured and flailed chordae tendinea of the prolapsed anterior leaflet, and significant mitral regurgitation with a regurgitation volume of 50ml. A supplementary finding was tricuspid regurgitation of borderline hemodynamic significance (Figure 1).

According to the current guidelines [9], a surgical procedure was indicated. Under extracorporeal circulation, as a minimally invasive cardiac surgery, the patient underwent mitral valve replacement by bioprosthesis, tricuspid valve annuloplasty with a ring and LAA occlusion by Gore stitch. The TEE performed during the surgery showed normal functioning of the mitral prosthesis and the tricuspid valve. LV EF was normal. LAA was without any residual leaks after occlusion. Effective warfarinization was set up aiming for an international normalized ratio (INR) between 2 – 2.5.

**Fig. 2:**
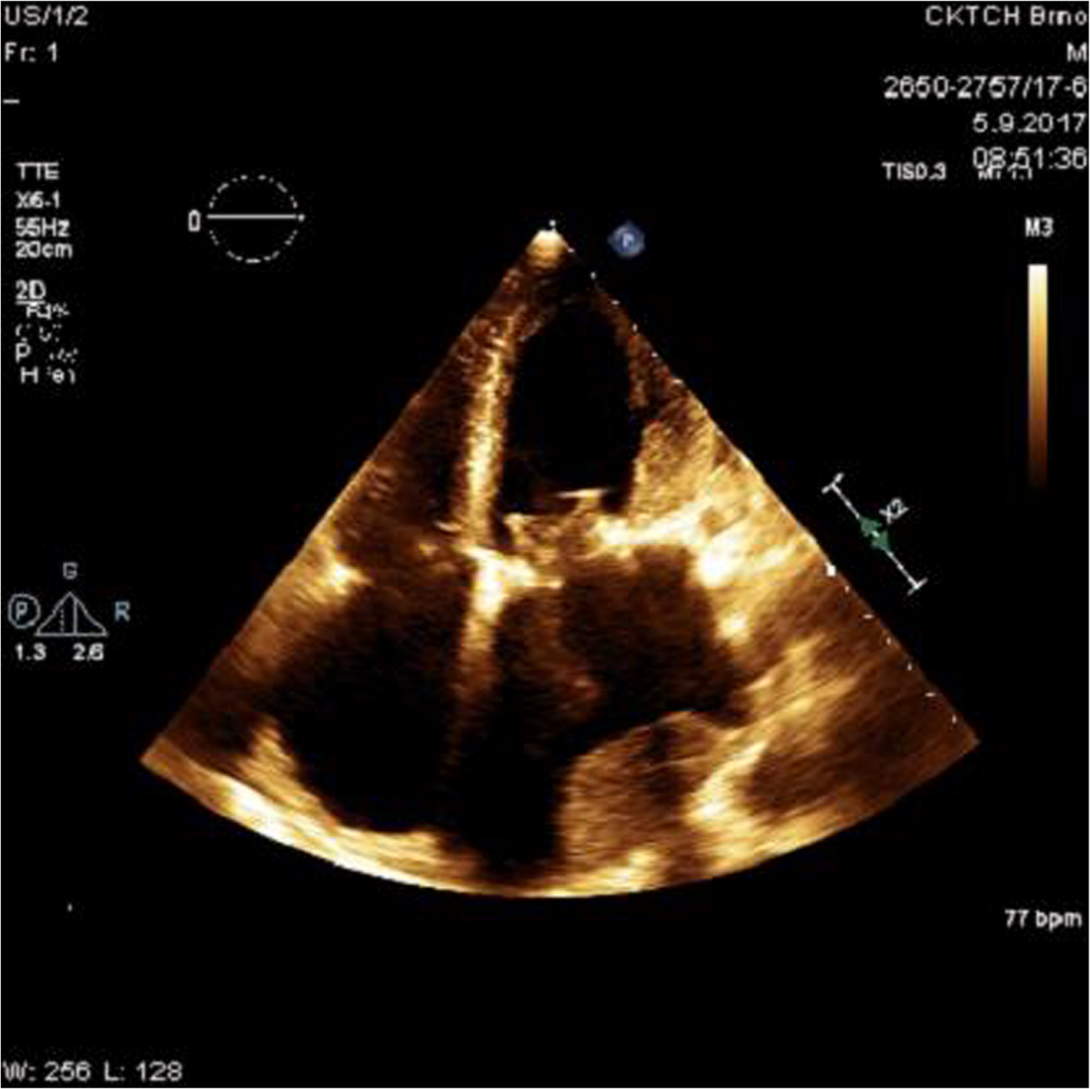
Echocardiogram 3 months post-surgery An apical four-chamber view showing an unknown left atrial mass attached to the posterior wall of the left atrium

**Fig. 3:**
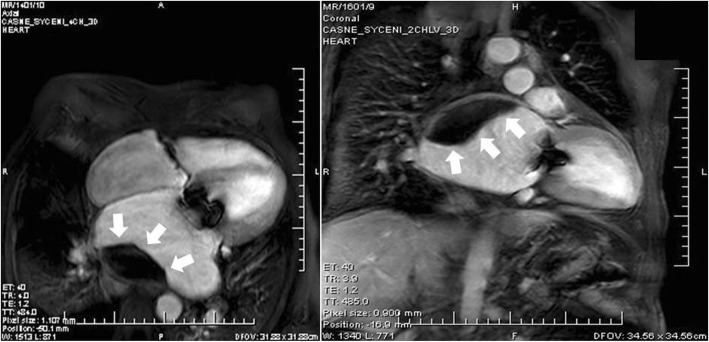
CMRI 3 months post-surgery CMRI – early gadolinium images, an apical four-(left figure) and two-(right figure) chamber view showing extreme dilatation of the LA with a large sessile thrombus (white arrows)

After 3 months post-surgery, the patient came in for his regular check-up, without any cardiac symptoms and with effective INR (2.13). A large sessile intra-atrial mass on the posterior wall of the giant LA was detected by TTE (Figure 2). The echocardiograph could not exclude the suspicion of mitral bioprosthesis dysfunction due to limited leaflet opening and increased LA/LV maximal gradient from the postoperative values from approximately 2-3 mmHg to 7-8 mmHg. Within a differential diagnostic procedure to differentiate possible thrombus from other intra-atrial masses (especially myxomas), CMRI was performed according to the standard protocol using a 1.5T scanner (Ingenia, Philips Medical Systems, Best, The Netherlands). Early gadolinium enhancement (EGE) images in both long-axis views and short axis views were acquired 1 minute after an intravenous bolus of 0.2 mmol/kg of the gadolinium-based contrast agent gadobutrol (Gadovist, Bayer-Schering Pharma, Germany). Extreme dilatation of LA (91x84mm) and a large sessile thrombus (60x30mm) on the posterior wall of the LA were confirmed by EGE images [10] (Figure 3).

Due to lasting suspicion for mitral valve dysfunction, i.e. suspected incomplete opening of the leaflets and increased LA/LV maximal gradient from 2-3 mmHg (postoperatively) to 7-8 mmHg, the patient was indicated for another cardiac surgery and concomitant thrombectomy was planned. During the surgery, mitral valve bioprosthesis function was verified as normal and the thrombus on the posterior wall of the LA was extracted and histologically verified. The patient was without any clinical or laboratory signs of inflammation and the thrombus did not contain any bacteria. Genetic mutations such as Leiden G1691A and prothrombin G20210A were tested for and found to be negative. The following TTE performed 24 hours after the thrombectomy showed only moderate smoke in the LA (reviewed as 2 out of 4 on the SEC Scale - Smoke EchoContrast) [11] and excluded the presence of a thrombus. Effective warfarinization was set up and according to the current guidelines, acetylsalicylic acid was added to VKA therapy for potentiation of anticoagulant therapy [9]. At the scheduled check-up 6 months after the first surgery, the patient was again in good clinical condition, with effective INR (2.49), but a recurrent thrombus (60x25mm) was again identified in the gigantic LA using echocardiography. The function of mitral bioprosthesis was good. As the patient did not shown any signs of embolic events or cardiac decompensation, a conservative approach was recommended and clopidogrel was added to the VKA instead of the previous acetylsalicylic acid. The target INR was increased to 2.5 – 3.

After this second recurrence, complex screening for potential causes was initiated. The patient was closely examined by a haematologist for hypercoagulable conditions such as the presence of immunoglobulin G and M (IgG and IgM) against cardiolipin, glycoproteins, antithrombin, fibrinogen, homocysteine – all of these markers were negative. Platelet function was assessed using a platelet function analyser with collagen, epinephrine and adenosine diphosphate and turned out to be normal. Screening for potential hematooncological malignancy also came out negative. Further oncological screening included computed tomography of the chest and an endoscopic examination of the colon (colonoscopy), as well as plasmatic oncomarker screening and there was no sign of any oncological process.

**Fig. 4:**
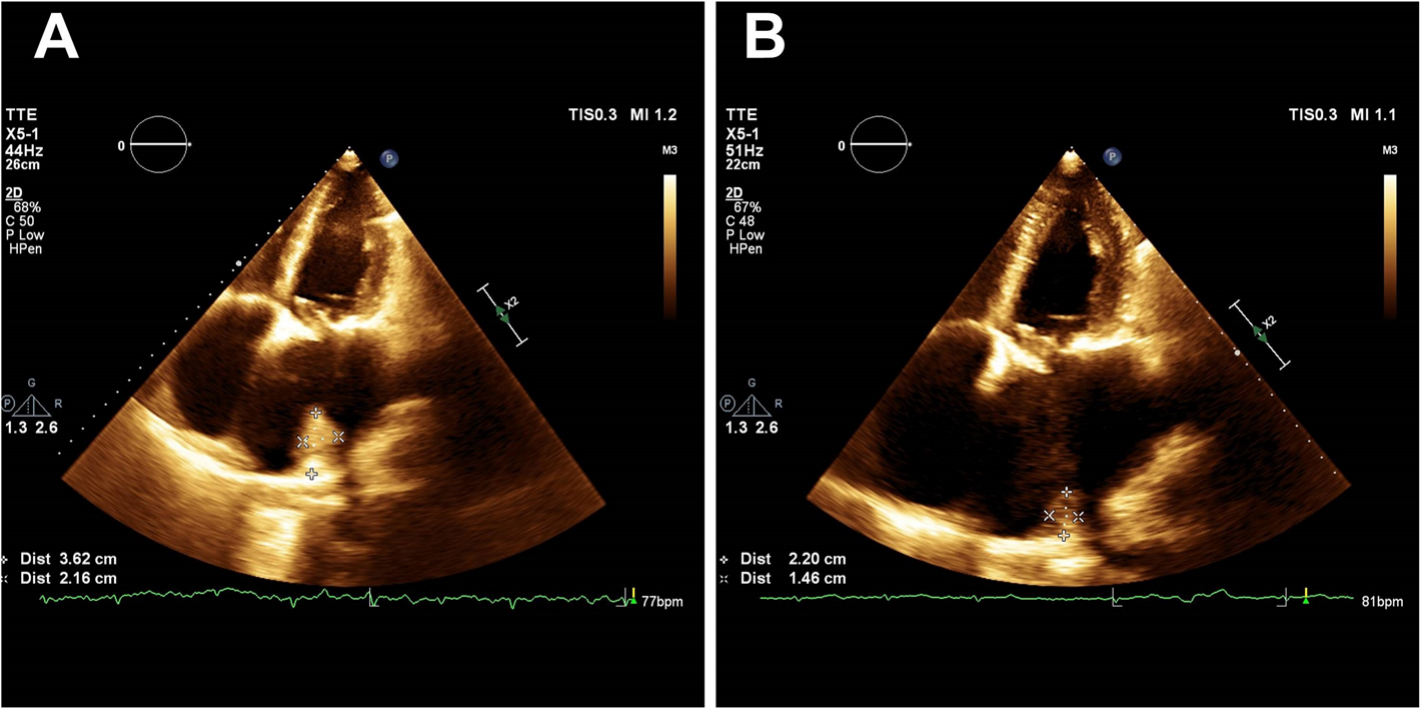
Echocardiograms 18 and 21 months post-surgery The apical four-chamber views showing a partially resolved thrombus attached to the posterior wall of the left atrium during the visits at 18 months (**A**) and 21 months (**B**) postsurgery

Nine months after the initial cardiac surgery, the combination of clopidogrel and increased anticoagulant therapy (INR 2.89) still had not resolved the thrombus that was verified and quantified by the CMRI. The left atrial size remained approximately the same (93x79mm) and the size of the recurrent thrombus was 90x30mm. The function of mitral bioprosthesis remained normal and the patient was in good clinical condition. A conservative approach was advised as there were no signs of embolic events or cardiac decompensation. The therapy was actually reduced to VKA monotherapy due to patient-referred excessive epistaxis, subcutaneous hematoma formation and pruritus. As long as these bleeding side effects continued to occasionally occur even after the therapy was reduced and laboratory tests furthermore revealed signs of the precipitating hepatopathy, VKA was discontinued after another 6 months from the previous visit (i.e. 15 months after the initial surgery). As all of the guideline-directed therapies had already been applied, combination therapy using rivaroxaban together with clopidogrel was initiated in order to keep effective anticoagulation of the patient and in order to try to dissolve the thrombus. During the last two follow-up visits that occurred after another 3 and 6 months from the last therapy adjustment (i.e. approximately 18 and 21 months after the initial surgery), the patient was still in good clinical condition, without any embolic events and without any signs of clinically relevant bleeding. Subsequent thrombus measurement by echocardiography finally showed a significant gradual decrease in size to 22x36mm and to 14x22mm, respectively (Figure 4A and 4B). The tailored therapy was maintained without any changes and the patient has been invited for a regular check-up in another 12 months.

## Discussion and Conclusions

The case described a male patient with non-rheumatic mitral valve regurgitation with gigantic LA and permanent AF who, after surgery on his mitral valve, developed a recurrent sessile thrombus on the posterior wall of the LA despite effective anticoagulation therapy. A suspected mass in the heart chambers can be accurately verified by echocardiography, contrast echo or CMRI. In patients with AF, CMRI has been proven to be a favourable diagnostic technique for the detection and assessment of LA/LAA thrombi [8]. Among the imaging sequences evaluated, EGE had the highest sensitivity, specificity, and diagnostic accuracy. On the other hand, echocardiography, and especially 3D echocardiography or contrast echocardiography, may enable better discrimination between the thrombi (usually located in the LLA having a wider base) and the most common cardiac tumours, e.g. myxomas (whose bases are narrower and that mostly arise from interatrial septum), depending on the location of the unknown intra-atrial mass [7, 12]. A combination of more methods may be reasonable, especially if the unknown masses are located atypically. Specifically, in the unique case of our patient in whom the thrombus originated from the posterior wall of the LA, a differential diagnosis by echocardiography was challenging and CMRI enabled a more precise diagnosis of the intra-atrial mass.

It is well known that AF is the most common sustained cardiac arrhythmia and predisposes the patient to an increased risk of thromboembolic events, with the most common location of thrombi being in the LAA [13]. Dilatation of the LA is associated with disturbances of blood flow/blood stasis and the formation of a thrombus. This results in a risk of thromboembolism which gradually increases with LA dimension, independent of the administration of anticoagulants [3]. This may be one of the contributing factors for the development of the thrombus in the described case, as the patient’s LA was gigantic.

Another aspect of the case is the fact that the thrombus appeared in the patient post mitral valve surgery. Prior to the surgery, he already had long standing AF (15 years) and probably also a gigantic LA and yet had developed no thrombus or thromboembolic event. It may be hypothesized that either mitral regurgitation itself prevented the patient from developing a thrombus as potential micro-emboli were dissolved or washed out from the LA during the regurgitation jet, or that during the cardiac surgery itself, the endocardium was somehow damaged, causing it to lose its anticoagulation potential, which led to the formation of the recurrent thrombus. The first hypothesis contrasts with the fact that mitral regurgitation itself is considered a procoagulation factor. The second hypothesis is supported by the fact that the posterior wall of the LA is where surgical devices may be stored during the operation [personal communication with surgeons] and furthermore, a similar case of thrombus development at the site of a previous LA myxoma operation has recently been described in literature [14]. In the current case, both factors may have contributed to the development of the thrombus.

However, it should be questioned why the thrombus still persisted in the gigantic LA even though standard therapy using VKA was administered. One of the contributing factors may be its location; it was reported by Silaruks et al. that no change in thrombus size despite effective anticoagulant therapy was observed when a thrombus was located in the body of the LA rather than the LAA [15]. According to the current American Heart Association (AHA) guidelines, our patient with permanent AF underwent mitral valve replacement by bioprosthesis for non-rheumatic mitral regurgitation and was subsequently treated with VKA. Three months after the surgery, our patient had a thrombus in the LA that developed despite effective VKA treatment and thus the therapy was enhanced by the addition of ASA [9], which also did not lead to the resolution of the thrombus. The treatment was then switched to clopidogrel, which still did not lead to a satisfactory resolution of the thrombus. Contrary to current AHA guidelines, which do not recommend NOAC administration in patients with biological heart valves, there have been several cases of patients with thrombi in the enlarged LA or LAA treated with NOAC described in the current literature in whose treatment with NOAC resulted in the dissolution of thrombi [16–18]. In our case, NOAC (specifically rivaroxaban) was used “off-label” and even combined with clopidogrel, which finally lead to the partial dissolution of the thrombus. NOACs provide some advantages over VKA with a faster onset, fewer drug interactions, and a fixed dose without the need for coagulation monitoring [19]. Nevertheless, NOACs are not recommended in non-rheumatic mitral stenosis because of a lack of evidence from clinical trials as all contemporary trials comparing NOAC with warfarin have excluded patients with clinically significant mitral stenosis [19]. Even though NOAC use cannot be widely recommended, in our case it lead to partial thrombus resolution, which provides a new potential way for the treatment of patients with previously unsolvable thrombi.

Despite effective anticoagulation therapy with VKA strengthened by ASA or clopidogrel, this case described a male patient with nonrheumatic mitral valve regurgitation with a gigantic LA and permanent AF who still developed a recurrent sessile thrombus after mitral valve surgery on the posterior wall of the LA. The thrombus was finally partially resolved after switching from VKA/ASA/clopidogrel to a combination of rivaroxaban and clopidogrel. Pathophysiological events leading to the formation of a thrombus are highly complex and potentially include endocardium damage caused during surgery, changes in blood flow caused by mitral valve repair and still present LA enlargement and other potential unrecognized alterations in the coagulation cascade undetectable using standard and special haematological approaches. CMRI was shown to be beneficial in providing a more precise description of the intra-atrial masses located on the posterior LA wall as compared to a standard echocardiographic examination, and thus may be helpful in the initial differential diagnostics of such atypically located intra-atrial masses. This case demonstrates that each patient requires a tailored therapy and personal approach, even beyond the current guidelines.

## Data Availability

Data sharing is not applicable to this article as no datasets were generated or analysed during the current study.

## Abbreviations

3D: Three-dimensional
AF: Atrial fibrillation
AHA: American Heart Association
ASA: Acetylsalicylic acid
cMRI: Cardiac magnetic resonance
EF: Ejection fraction
fT4: Free T4
INR: International normalized ratio
LA: Left atrium
LAA: Left atrial appendage
LV: Left ventricle
NOAC: Novel anticoagulants
TEE: Transesophageal echocardiography
TSH: Thyroid stimulating hormone
TTE: Transthoracic echocardiography
VKA: Vitamin-K antagonists
